# An ab initio study on noble gas inserted halogenated acetylene: HNgCCX (Ng = Kr and Xe; X = halogen)

**DOI:** 10.1038/s41598-017-10786-0

**Published:** 2017-08-31

**Authors:** Zhengguo Huang, Yuying Li, Xiaohong Wang

**Affiliations:** Tianjin Key Laboratory of Structure and Performance for Functional Molecules, Key Laboratory of Inorganic-Organic Hybrid Functional Materials Chemistry (Tianjin Normal University), Ministry of Education, College of Chemistry, Tianjin Normal University, Tianjin, 300387 People’s Republic of China

## Abstract

Although HNgCCX (Ng = Kr and Xe; X = F and Cl) have been identified in cryogenic matrices, similar Br and I analogues have not been prepared so far. In this paper, the nature of HNgCCX (Ng = Kr and Xe; X = F, Cl, Br and I) have been investigated by ab initio methods. The main characteristic absorption peak of HNgCCX is the *v*_H-Ng_, which decreases as X varies from F to I. Moreover, the H-Xe bond is stronger than the H-Kr bond. The *v*_C≡C_ and *v*_C-X_ exhibit red- and blue-shift characters, respectively, especially the C-X bond is abnormal blue-shift halogen bond. AIM results show that the H-Ng bond is essentially covalent bond and the covalent character of H-Xe bond is underestimated, and the trend of the covalent character is C-Cl > C-Br > C-F > C-I. Although HNgCCX is instable thermodynamically with respect to Ng + HCCX, it is kinetically stable with respect to the two-/three-body channels due to the relatively larger energy barriers. The three-body channels of HNgCCX is the main decomposition channel, and the kinetically stability of HXeCCX is more than its Kr analogues. This study is helpful for the preparation of new HNgCCX in cryogenic matrices.

## Introduction

The noble gases were regarded as to be chemically inert for a long time until the first xenon-containing molecule has been synthesized by Bartlett in 1962. Lots of noble-gas molecules, namely HNgY (Ng is a noble-gas atom, and Y represents a strongly electronegative atom or group), have been identified in cryogenic noble-gas matrices since the mid-1990s^1–14^. It is generally accepted that HNgY has partly ionic character and can be represented by (H-Ng)^+^Y^−^, where (H-Ng)^+^ is mainly covalent, whereas the interaction between (HNg)^+^ and Y^−^ is predominantly ionic. The structures, bonding interactions, spectra, and reaction mechanisms of the HNgY species have been topics of many studies, which enriched our understanding of noble-gas chemistry^15, 16^ for instance, the identification of the first neutral argon molecule (HArF)^17^ and halogen-free organic noble-gas molecules (HXeCCH, HXeCC, HKrCCH, etc.)^18–22^ are major highlights of the field. HNgY are not only remarkable for exploring the frontiers of chemical reactivity, but also are special in many other respects because of their relatively weak bonding and large dipole moments, which results in strongly enhanced effects of the environment, complexation and reactions. The studies on HNgY reveals photo-dissociation dynamics and atomic mobility in noble-gas solid matrices.

HXeCCH is the first hydrocarbon with an inserted noble-gas atom observed in cryogenic noble-gas matrices^18, 19, 23, 24^. Subsequently, many organic noble gas molecules have been investigated experimentally and theoretically, for example, HKrCCH^12, 13, 21, 22, 25^, HNgC_4_H (Ng = Kr and Xe)^26^, XNgCCX and XNgCCNgX (Ng = Kr, Ar; X = F, CI)^27^, HNgCCX and HCCNgX (Ng = Kr, and Xe; X = F and Cl)^4, 14^, and so on. Previous works proved that no HCCNgX were observed in cryogenic noble-gas matrices although there are two possible reactive sites in HCCX (H-C and C-X bonds) for the insertion of Ng atoms. Moreover, HNgCCX is expected to be higher stable than the corresponding HNgCCH due to the higher H-Ng stretching frequency. These studies intrigued our interests in the halogenated organic noble-gas molecule HNgCCX (Ng = Kr and Xe; X = F, Cl, Br and I).

Since HNgCCX (Ng = Kr and Xe; X = F and Cl) have been identified by cryogenic matrix isolation technique combined with quantum chemical calculations, similar Br and I analogues are expected to be enough stable to be characterized in cryogenic noble-gas matrices, however, no reports about HCCNgX (Ng = Kr and Xe; X = Br and I) are found so far. Therefore, we systematically investigate HNgCCX (Ng = Kr and Xe; X = F, Cl, Br and I) in this paper. The focuses of our attention are whether HNgCCX (Ng = Kr and Xe; X = Br and I) are enough stable to be prepared and identified experimentally, and what are the differences in the nature of bonding and spectra among HNgCCX molecules? Furthermore, we hope that this study will be helpful for the preparation of HNgCCX (Ng = Kr and Xe; X = Br and I) molecules in cryogenic noble-gas matrices.

## Results and Discussions

### Structures

**Figure 1:**
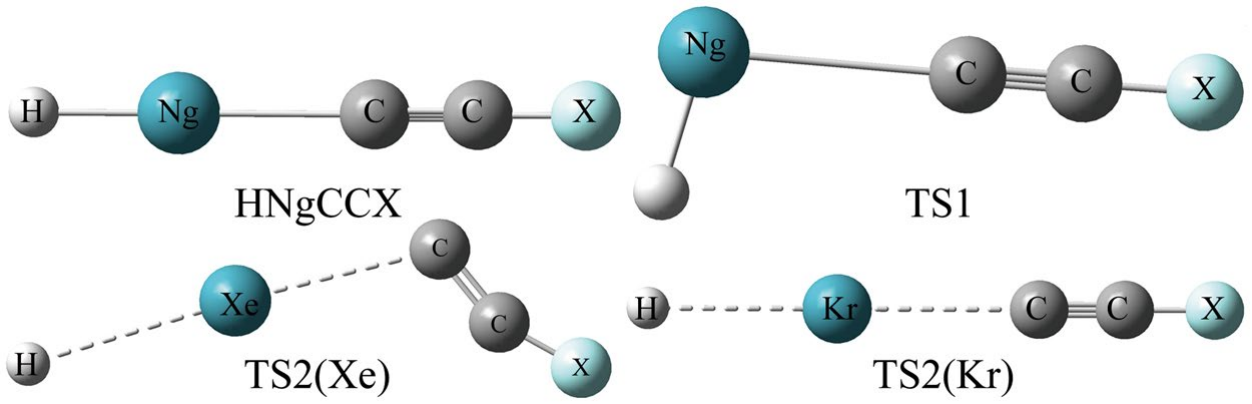
The models of HNgCCX (Ng = Kr and Xe; X = F, Cl, Br and I) and their transition states (TS1 and TS2) for the two-/three-body channels.

The models of HNgCCX (Ng = Kr and Xe; X = F, Cl, Br and I) and the concerning transition states (TS1 and TS2) for the two-body and three-body channels were presented in Fig. 1, and structural parameters of optimized HNgCCX were presented in Table 1. HNgCCF and HNgCCCl (Ng = Kr and Xe) have been already prepared and identified in low-temperature noble-gas matrices^4, 14^, while HNgCCBr and HNgCCI (Ng = Kr and Xe) were studied firstly in the present paper. It can be learned from Table 1 that the optimized parameters of both HNgCCF and HNgCCCl (Ng = Kr and Xe) are very close to previous works which performed at the MP2 and CCSD(T) levels^4, 14^. All HNgCCX are linear geometries with C_∞v_ symmetry. As shown in Table 1, the R_C-X_ of HNgCCX is lengthened as X varies from F to I, which is mainly due to the increasing radius of halogen atom. Moreover, the replacement of Ng atoms has little influence on the R_C-X_ since the R_C-X_ of HKrCCX is slightly longer than that of HXeCCX. Similarly, the R_C≡C_ of HKrCCX is affected little by the replacement of Ng atoms. However, compared with the free HCCX molecule, both the R_C-X_ and R_C≡C_ are lengthened simultaneously, which indicates that the insertion of Ng atom into C-H bond activate the C≡C and C-X bonds to some extent. In the same way, both H-Ng and Ng-C bonds of HNgCCX are weakened slightly as X varies from F to I due to the lengthened bond lengths, which indicates that the replacement of halogen atoms has little influence on them. Moreover, both H-Xe and Xe-C bonds of HXeCCX are larger than the corresponding bonds in its Kr counterpart, which mainly is attributed to the different radii of noble gas atoms, and another plausible reason is that both H-Xe and Xe-C bonds of HXeCCX are stronger than the corresponding bonds in its Kr counterpart.

**Table 1:** The bond lengths (in Å) of HNgCCX (Ng = Kr and Xe; X = F, Cl, Br and I) and the concerning precursors calculated at the MP2(full)/aug-cc-pVTZ-PP/aug-cc-pVTZ level.

	HKrCCF	HKrCCCl	HKrCCBr	HKrCCI	HXeCCF	HXeCCCl	HXeCCBr	HXeCCI
R_H-Ng_	1.554	1.561	1.562	1.564	1.722	1.725	1.725	1.727
R_Ng-C_	2.221	2.223	2.222	2.223	2.327	2.330	2.331	2.332
R_C≡C_	1.222	1.229	1.230	1.232	1.223	1.230	1.231	1.233
R_C-X_	1.286	1.643	1.787	1.984	1.284	1.641	1.784	1.983
ΔR_H-Ng_	0.064	0.071	0.072	0.074	0.092	0.095	0.095	0.097
ΔR_Ng-C_	0.301	0.303	0.302	0.303	0.267	0.270	0.271	0.272
ΔR_C-X_	−0.104	−0.097	−0.103	−0.096	−0.106	−0.099	−0.106	−0.097
	**HCCF**	**HCCCl**	**HCCBr**	**HCCI**				
R_H-C_	1.053	1.054	1.054	1.056				
R_C≡C_	1.201	1.209	1.210	1.213				
R_C-X_	1.276	1.633	1.776	1.976				

It is noteworthy that the relatively strength of C-X bond containing different halogen atom cannot be estimated directly by their lengths. Likewise, although the H-Kr (or Kr-C) bond in HKrCCX is shorter than the H-Xe (or Xe-C) bond of its Xe counterpart, which does not mean that the former must be stronger than the latter because of the different radii of noble gas atoms. Therefore, in order to compare these bonds containing different atoms, one structural parameter Δ*R* was defined as follow^28, 29^:

1 $${\rm{\Delta }}{R}_{{\rm{A}} \mbox{-} {\rm{B}}}={R}_{{\rm{A}} \mbox{-} {\rm{B}}}-{R}_{{\rm{A}}}\,-\,{R}_{{\rm{B}}}$$

where *R*_A-B_ is the bond length of A-B bond, *R*_A_ and *R*_B_ are the covalent radii of A and B atoms^30^, respectively. The smaller Δ*R*_A-B_ is, the stronger the interaction is, and vice versa. The calculated Δ*R*_H-Ng_, Δ*R*_Ng-C_ and Δ*R*_C-X_ were presented in Table 1 as well. As shown in Table 1, the negative Δ*R*_C-X_ indicates that the C-X bond should be stronger than the typical C-X bond, whereas both Δ*R*_H-Ng_ and Δ*R*_Ng-C_ are positive, which means these bonds are weaker than the typical H-Ng (or Ng-C) bond. The Δ*R*_Kr-C_ of HKrCCX is about 0.30 Å and is larger than the Δ*R*_Xe-C_ (~0.27 Å) of HXeCCX, which demonstrates that the Kr-C bond may be weaker than the Xe-C bond. Unlike Δ*R*_Ng-C_, the Δ*R*_H-Kr_ of HKrCCX is within the range of 0.064~0.074 Å, which is smaller than the Δ*R*_H-Xe_ (0.092~0.097 Å) of HXeCCX, so the H-Kr bond seems to be stronger slightly than the H-Xe bond. To be clear, Δ*R* is not the unique criterion for the evaluation of these bonds, but provide the preliminary information on the strengths of bonds, and the nature of these bonds will be discussed later.

### Frequencies

IR spectroscopy is one of the most commonly used tools to characterize active species in low-temperature matrices, and the vibrational fingerprints can provide important information for molecular identification and structural analysis. The vibrational frequencies of HNgCCX (Ng = Kr and Xe; X = F, Cl, Br and I) and the concerning precursors calculated at the MP2 level were presented in Table 2. The H-Ng stretching vibrational mode is the characteristic absorption peak of HNgCCX because it has the highest intensity and is very likely to be observed experimentally. As shown in Table 2, the calculated H-Kr (*v*_H-Kr_) harmonic vibration frequencies of HKrCCF and HKrCCCl are 1687.1 and 1651.5 cm^−1^, respectively, which are very close to the previous theoretical works obtained at the MP2 and CCSD(T) levels^4, 14^, however, they are larger than the experimental values of *v*_H-Kr_ due to the remarkable anharmonicity of the H-Kr stretching vibrational mode. Similar to the H-Kr stretching vibrational mode, the calculated H-Xe (*v*_H-Xe_) of HXeCCF and HXeCCCl are 1760.2 and 1740.6 cm^−1^, respectively, which keep in line with the previous theoretical result, and behaves strong anharmonic character as well since they are obviously larger than those experimental data in solid noble gas matrices^4, 14^. Moreover, the *v*_H-Ng_ decreases as X varies from F to I, which reflects the fact of the decreasing H-Ng bond strength from one aspect. In addition, the *v*_H-Xe_ of HXeCCX is about 100 cm^−1^ higher than the *v*_H-Kr_ of HKrCCX, which reveals that the H-Xe bond should be stronger than the H-Kr bond, which is consistent with the structural results above.

**Table 2:** The selected vibrational frequencies (in cm^−1^) of HNgCCX (Ng = Kr and Xe; X = F, Cl, Br and I) and the concerning precursors calculated at the MP2(full)/aug-cc-pVTZ-PP/aug-cc-pVTZ level^a^.

	*v*_H-Kr_	*v*_Kr-C_	*v*_C≡C_	*v*_C-X_		*v*_H-Xe_	*v*_Xe-C_	*v*_C≡C_	*v*_C-X_
HKrCCF	1687.1 (1931)	262.3 (147)	2204.5 (198)	1090.4 (32)	HXeCCF	1760.2 (1161)	244.9 (124)	2201.3 (234)	1094.2 (22)
HKrCCCl	1651.5 (2425)	229.1 (116)	2087.0 (78)	809.6 (28)	HXeCCCl	1740.6 (1418)	209.8 (94)	2081.7 (99)	811.7 (28)
HKrCCBr	1640.5 (2621)	189.0 (74)	2064.1 (53)	686.6 (97)	HXeCCBr	1739.3 (1524)	168.3 (57)	2060.8 (69)	686.9 (90)
HKrCCI	1630 (2922)	169.3 (54)	2044.2 (29)	625.8 (192)	HXeCCI	1730.6 (1680)	147.9 (40)	2040.7 (41)	623 (175)
	***v***_**C-H**_		***v***_**C≡C**_	***v***_**C-X**_					
HCCF	3542.6 (97)		2267 (131)	1078.4 (76)					
HCCCl	3530.1 (92)		2127.6 (37)	771.9 (9)					
HCCBr	3523.3 (91)		2101.7 (23)	621.6 (1)					
HCCI	3509.4 (88)		2072.9 (9)	543.4 (1)					

^a^Numbers given in parentheses are the respective calculated IR intensities (in km·mol^−1^).

The calculated C≡C stretching vibrational mode (*v*_C≡C_) of HKrCCF and HXeCCF are 2204.5 and 2201.3 cm^−1^, respectively, which are close to the experimental values (2177 and 2171 cm^−1^) in solid noble gas matrices and exhibit relatively weak anharmonicities^4^, meanwhile they show red shifts of about 60 cm^−1^ with respect to the precursor HCCF. Similarly, the calculated *v*_C≡C_ of HKrCCCl and HXeCCCl are 2087.0 and 2081.7 cm^−1^, respectively, which show red shifts of about 40 cm^−1^ with respect to HCCCl and are close to the experimental values (2070.7 and 2077.4 cm^−1^) in noble gas matrices as well^14^. The calculated *v*_C≡C_ of other HNgCCX (Ng = Kr and Xe; X = Br and I) are more 100 cm^−1^ lower than that of HNgCCF, and demonstrates red shifts of tens wavenumbers as well. As shown in Table 2, the C≡C bond of HNgCCF is expected to be the strongest due to the highest *v*_C≡C_, and is weakened as X varies from F to I, moreover, the activation of C≡C bond by the insertion of Ng atoms into the H-C bonds of HCCX is supported by the red shift of *v*_C≡C_ as well.

The calculated C-F harmonic vibration frequencies (*v*_C-F_) of both HKrCCF and HXeCCF are 1090.4 and 1094.2 cm^−1^, respectively, which are close to the experimental values (1051 and 1054 cm^−1^) in solid matrices and show weak anharmonicities as well^4^. The calculated *v*_C-Cl_ of HNgCCCl (X = Kr and Xe) are about 810 cm^−1^, which is consistent with previous theoretical results^14^, however, no experimental data were reported and one possible reason is that such C-Cl stretching vibration mode is weak. Unlike the *v*_C≡C_, the C-X stretching vibrational mode (*v*_C-X_) exhibits blue shift of tens wavenumbers with respect to the precursor HCCX. As discussed above, the C-X bond is lengthened during the insertion of Ng atoms into the H-C bonds of HCCX, whereas the blue-shift halogen bond usually corresponds to the shortening of C-X bond, so the C-X bond is against the “normal” blue-shift halogen bond, and the reason needs to be studied further. As shown in Table 2, the *v*_C-X_ of HKrCCX is close to that of HXeCCX, but decreases as X varies from F to I, which reveals that the *v*_C-X_ is mainly affected by the replacement of halogen atoms rather than the replacement of noble-gas atoms. Moreover, the blue-shift value (Δ*v*_C-X_) increases as X varies from F to I. Another stretching vibrational mode (*v*_Ng-C_), in principle, can be used to characterize and identify HNgCCX in experiments, but the *v*_Ng-C_ is beyond the limit of infrared spectrometer, which makes the identification of HNgCCX difficult to some extent. In addition, the decreasing of *v*_Ng-C_ means that the H-Ng bond is weakened as X varies from F to I.

### AIM and FBO analyses

**Figure 2:**
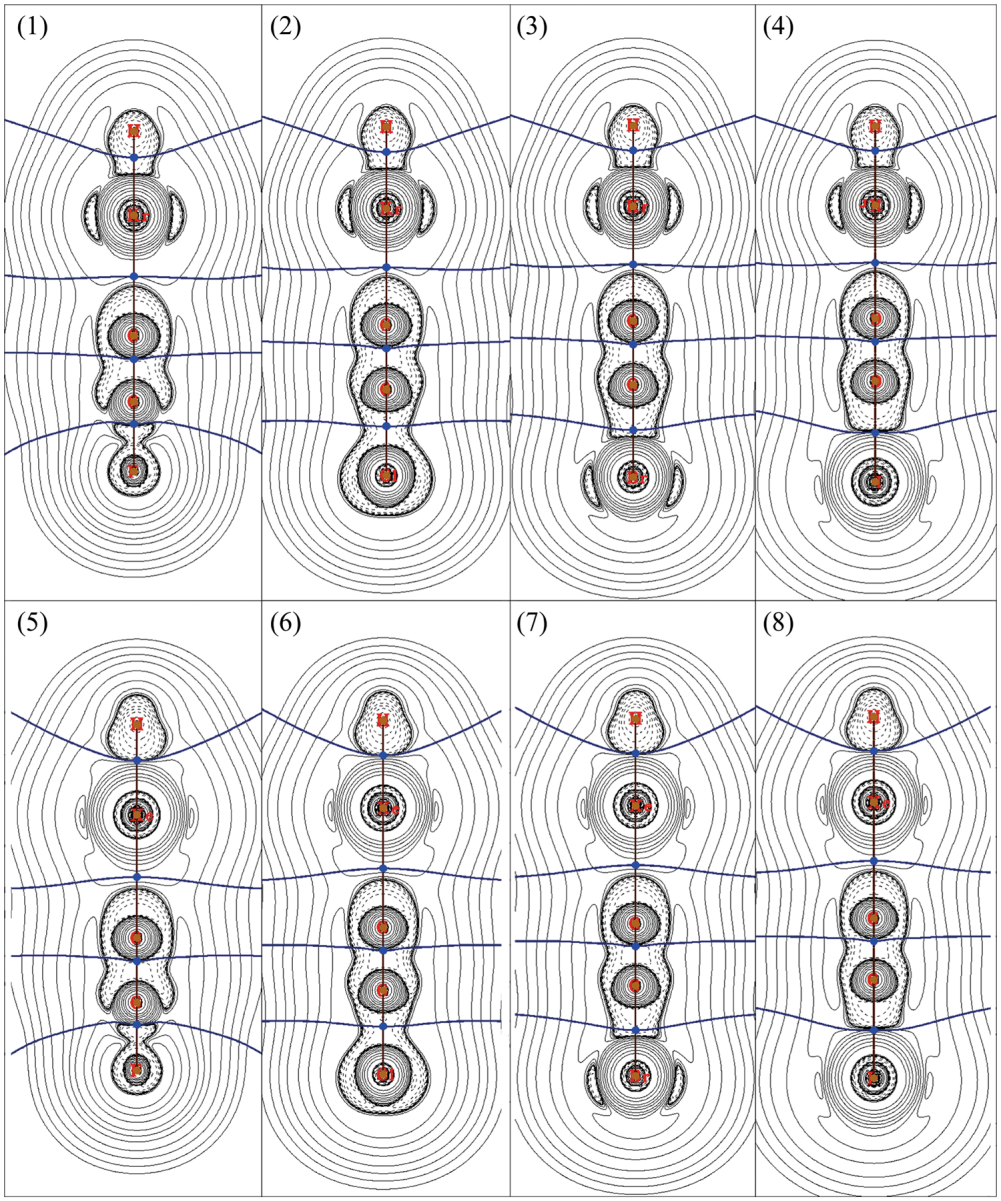
Contour line diagrams of ∇^2^ρ_b_ for HNgCCX (Ng = Kr and Xe; X = F, Cl, Br and I), obtained by MP2(full) method with all-electron basis sets. (1) HKrCCF; (2) HKrCCCl; (3) HKrCCBr; (4) HKrCCI; (5) HXeCCF; (6) HXeCCCl; (7) HXeCCBr; (8) HXeCCI. Dashed lines indicate areas of charge concentration (∇^2^ρ_b_ < 0) while solid lines show areas of charge depletion (∇^2^ρ_b_ > 0). The bold brown solid lines connecting the atomic nuclei are the bond paths and the solid blue lines separating the atomic nuclei indicate the zero-flux surfaces in the molecular plane. The crossing points of the bond paths and zero-flux surfaces are the bond critical points (BCP).

To understand the nature of bonds in HNgCCX (Ng = Kr and Xe; X = F, Cl, Br and I), AIM analyses have been performed and the contour line diagrams of Laplacian of electron density (∇^2^ρ_b_) for HNgCCX were illustrated in Fig. 2, and the results were presented in Table 3. As shown in Fig. 2, no inner shell structure can be found for H atom since it just has 1s valence shell, the presence of dashed isosurfaces around H atom illustrates a region of charge concentration, and the valence shell region is strongly distorted towards accumulated charge concentration in the bonding region. On the contrary, the Ng atom has a region of charge depletes along the H-Ng bond line direction. Similarly, the C atom of CCX groups has a region of charge concentration towards the Ng atom, while the Ng atom has a region of charge depletion region along the Ng-C bond line direction. Therefore, there is charge transfers from the Ng to the H and CCX group, and both the H-Ng and Ng-C bonds illustrate closed-shell interaction characters. The BCP of H-Kr bond locates at the charge concentration region around the H atom, which is different from that of H-Xe bond since the latter locates at the boundary between the charge concentration region and the charge depletion region. Such difference leads to an underestimation of the covalent character of H-Xe bond by AIM parameters to some extent. Moreover, such difference makes the directly comparison between H-Xe and H-Kr bonds unavailable. Of course, such difference does not affect the comparisons among the H-Kr bonds of HKrCCX, so the H-Xe bonds of HXeCCX. Unlike the H-Ng bond, the Ng-C bond illustrates typical closed-shell interaction characters. In addition, the Kr atom has a charge concentration region which shows maxima in the perpendicular direction to the bonding line, however, such charge concentration region cannot be found for the Xe atom, which is due to more charge transfer happened in the Xe system.

**Table 3:** The AIM parameters (in a.u.) of HNgCCX (Ng = Kr and Xe; X = F, Cl, Br and I) carried out using the MP2(full) method with all-electron basis sets.

molecules	BCP	ρ_b_	∇^2^ρ_b_	G_b_	V_b_	|V_b_|/G_b_	H_b_
HKrCCF	H-Kr	0.1689	−0.3499	0.0409	−0.1692	4.1412	−0.1283
	Kr-C	0.0891	0.0811	0.0496	−0.0790	1.5917	−0.0294
	C-C	0.3769	−0.3736	0.5606	−1.2147	2.1666	−0.6540
	C-F	0.2988	0.5234	0.5847	−1.0385	1.7762	−0.4538
HKrCCCl	H-Kr	0.1669	−0.3419	0.0400	−0.1656	4.1347	−0.1255
	Kr-C	0.0894	0.0802	0.0496	−0.0791	1.5954	−0.0295
	C-C	0.3904	−0.7311	0.4971	−1.1770	2.3677	−0.6799
	C-Cl	0.2532	−0.6233	0.0882	−0.3322	3.7674	−0.2440
HKrCCBr	H-Kr	0.1663	−0.3397	0.0398	−0.1645	4.1334	−0.1247
	Kr-C	0.0896	0.0799	0.0497	−0.0794	1.5982	−0.0297
	C-C	0.3934	−0.8164	0.4800	−1.1641	2.4252	−0.6841
	C-Br	0.2052	−0.3130	0.0913	−0.2609	2.8567	−0.1696
HKrCCI	H-Kr	0.1658	−0.3376	0.0397	−0.1637	4.1283	−0.1241
	Kr-C	0.0896	0.0799	0.0496	−0.0793	1.5974	−0.0297
	C-C	0.3967	−0.9071	0.4624	−1.1516	2.4904	−0.6892
	C-I	0.1452	0.1799	0.1315	−0.2180	1.6579	−0.0865
HXeCCF	H-Xe	0.1286	0.0062	0.0838	−0.1661	1.9816	−0.0823
	Xe-C	0.0779	0.1294	0.0591	−0.0859	1.4529	−0.0268
	C-C	0.3774	−0.4035	0.5542	−1.2093	2.1820	−0.6551
	C-F	0.2998	0.5310	0.5882	−1.0437	1.7743	−0.4555
HXeCCCl	H-Xe	0.1280	0.0028	0.0822	−0.1638	1.9913	−0.0815
	Xe-C	0.0784	0.1239	0.0583	−0.0857	1.4691	−0.0274
	C-C	0.3905	−0.7549	0.4890	−1.1668	2.3859	−0.6778
	C-Cl	0.2543	−0.6296	0.0890	−0.3355	3.7675	−0.2464
HXeCCBr	H-Xe	0.1281	0.0026	0.0823	−0.1640	1.9920	−0.0817
	Xe-C	0.0790	0.1205	0.0581	−0.0860	1.4810	−0.0279
	C-C	0.3937	−0.8393	0.4725	−1.1548	2.4441	−0.6823
	C-Br	0.2062	−0.3152	0.0928	−0.2645	2.8488	−0.1716
HXeCCI	H-Xe	0.1277	0.0008	0.0814	−0.1626	1.9977	−0.0812
	Xe-C	0.0781	0.1271	0.0588	−0.0858	1.4596	−0.0270
	C-C	0.3970	−0.9228	0.4563	−1.1432	2.5056	−0.6870
	C-I	0.1448	0.1953	0.1344	−0.2200	1.6367	−0.0856

As shown in Fig. 2, the charge concentration region around C≡C bond reveals that it is a typical covalent bond. The charge concentration region around the C-X (X = F and Cl) bond illustrates the covalent character as well, and the charge concentration region of the halogen atom decreases simultaneously as the X varies from Cl to I, which demonstrates a gradually transition from covalent bond to ionic bond. Therefore, the directly comparison among the C-X bonds involving different halogen atoms is unavailable because their concrete situations are different from each other. Of course, the comparison between the C-X bonds in HKrCCX and its Xe counterpart is still feasible due to the similar situations.

Some AIM descriptors at BCPs have been used widely to explore the nature of bonding interaction, including the electron density (ρ_b_) and its Laplacian (∇^2^ρ_b_), the total energy density (H_b_), the absolute ratio of potential and kinetics energy densities (|V_b_|/G_b_), and so on ref. 31. It is generally accepted that both H_b_ and ∇^2^ρ_b_ at the BCP allow the interaction to be characterized^31–34^: H_b_ < 0 and ∇^2^ρ_b_ < 0 indicates an accumulation of charge density at BCP and corresponds to covalent interaction between the interacting atoms; H_b_ > 0 and ∇^2^ρ_b_ > 0 indicates a depletion of charge density at BCP and corresponds to closed-shell interaction between the interacting atoms; H_b_ < 0 but ∇^2^ρ_b_ > 0 is indicative of partially covalent interactions. The |V_b_|/G_b_ ratio is another useful descriptor to discriminate interaction types: |V_b_|/G_b_ < 1 denotes pure closed-shell interaction; |V_b_|/G_b_ > 2 denotes pure covalent (open-shell) interaction; while 1 < |V_b_|/G_b_ < 2 denotes intermediate interaction^31^. As shown in Table 3, the C≡C bond has negative ∇^2^ρ_b_ and H_b_, and the |V_b_|/G_b_ ratio is larger than 2.0, which indicates that it is a typical covalent bond. Similarly, the H-Kr bond in HKrCCX (X = F, Cl, Br and I) has negative ∇^2^ρ_b_ and H_b_, and the |V_b_|/G_b_ ratio is larger than 4.0, which illustrates that the H-Kr bond seems to have more covalent character than the C≡C bond. For the BCP of the H-Xe bond, the H_b_ < 0 but ∇^2^ρ_b_ > 0 indicates intermediate interaction, however, the covalent character of the H-Xe bond is underestimated because the BCP locates at the boundary between the charge concentration region and the charge depletion region. Moreover, the |V_b_|/G_b_ ratio of H-Xe BCP is close to 2.0, which further reveals that the H-Xe bond is covalent bond rather than ionic bond. For the BCP of the C-X bond, the negative H_b_ and ∇^2^ρ_b_ of both C-Cl and C-Br bonds indicate that they are covalent bonds, while the C-F and C-I bonds are partial covalent bonds due to the negative H_b_ and positive ∇^2^ρ_b_. Moreover, the |V_b_|/G_b_ ratio of also reveals the transition trend of the covalent character of C-X bond: C-Cl > C-Br > C-F > C-I, which agree with the analyses about Fig. 2. According to Fig. 2, the Ng-C bond should be closed-shell interaction, the further analysis of Table 3 illustrates that the Ng-C bond belong to intermediate interaction due to the negative H_b_ and positive ∇^2^ρ_b_, which is also supported by the |V_b_|/G_b_ ratio. In summary, the HNgY (Y is a strongly electronegative atom or group) has been generally considered to (H-Ng)^+^Y^−^, where (H-Ng)^+^ is mainly covalent, whereas the interaction between (H-Ng)^+^ and Y^−^ is predominantly ionic, however, the AIM results here illustrates that the covalent character of the H-Xe bond is underestimated because the BCP locates at the boundary between the charge concentration region and the charge depletion region. To understand the bonds in HNgCCX (Ng = Kr and Xe; X = F, Cl, Br and I), electron localization function (ELF) analyses have been performed and the ELF color-filled map of HNgCCX were showed in Fig. S1 (see Supplementary information), and the results further confirm the AIM conclusion.

The Fuzzy bond order (FBO)^35, 36^ were calculated using MP2(full) method, and cc-pVTZ basis sets removing the diffusion basis functions were used to obtain reliable results. The calculated results were listed in Table 4. As shown in Table 4, the FBO of H-Kr bond is smaller than that of H-Xe bond, which indicates that the H-Kr bond is weaker than the H-Xe bond and is consistent with the results above. Similarly, the Kr-C bond is weaker than the Xe-C bond due to the smaller FBO. Unlike the H-Kr and Kr-C bonds, the C-C bond in HKrCCX is stronger that the corresponding one in HXeCCX, which demonstrates that the C≡C bond is more likely to be activated by the insertion of Xe atom. As X varies from F to I, the FBOs of H-Ng and Ng-C bonds decrease slightly, which illustrates that both the bonds are weakened and is in accord with the results above. The C-Cl bond in HNgCCX has the largest FBO value among all C-X bonds, which reveals that it is the strongest C-X bond, and the C-X bond is weakened as X varies from Cl to I due to the decreasing FBO, which is essentially in agreement with the AIM results, and the discrepancy is the C-F bond, its covalent character is between the C-Br and C-I bonds according to AIM results, but the FBO of C-Cl is larger than that of C-Br bond. With their FBO so close, the difference is negligible.

**Table 4:** The Fuzzy bond orders (FBO) of HNgCCX (Ng = Kr and Xe; X = F, Cl, Br and I) calculated at the MP2(full)/cc-pVTZ-PP/cc-pVTZ level.

FBO	H-Ng	Ng-C	C-C	C-X
HKrCCF	0.939	0.936	2.679	1.390
HKrCCCl	0.935	0.919	2.601	1.403
HKrCCBr	0.934	0.916	2.600	1.387
HKrCCI	0.932	0.909	2.580	1.359
HXeCCF	1.017	1.028	2.645	1.392
HXeCCCl	1.015	1.010	2.571	1.404
HXeCCBr	1.015	1.006	2.571	1.387
HXeCCI	1.013	0.998	2.553	1.357

### The thermodynamic and kinetic stabilities

Previous works on reaction mechanisms can provide reference and inspiration for the study on reaction mechanisms of HNgCCX^37–41^, which may cast light on the stabilities of HNgCCX from two aspects: thermodynamics and kinetics. It has been generally considered that there are two decomposition channels of HNgCCX, namely the two-body and three-body channels, corresponding to the different products are given below,

2 $${\rm{HNgCCX}}\to {\rm{HCCX}}+{\rm{Ng}}\,{\rm{\Delta }}{{\rm{E}}}_{2{\rm{B}}}={{\rm{E}}}_{{\rm{Ng}}}+{{\rm{E}}}_{{\rm{HCCX}}}\,-\,{{\rm{E}}}_{{\rm{HNgCCX}}}$$

3 $${\rm{HNgCCX}}\to {\rm{H}}+{\rm{Ng}}+{\rm{CCX}}\,{\rm{\Delta }}{{\rm{E}}}_{3{\rm{B}}}={{\rm{E}}}_{{\rm{H}}}+{{\rm{E}}}_{{\rm{Ng}}}+{{\rm{E}}}_{{\rm{CCX}}}-{{\rm{E}}}_{{\rm{HNgCCX}}}$$

The two-body channel (2) corresponds to the product of HY and Ng, while the three-body channel (3) results in the final products of neutral H, Ng atom and the neutral Y group. The single-point energies of the reactant, products and the concerning transition states (TS) for both channels were calculated at the MP2(full) and CCSD(T) levels, in which zero-point vibrational energy (ZPVE) correction was not considered by CCSD(T) method because it is very time-consuming, then the decomposition energies and the corresponding energy barriers were calculated at the same levels, and the results were listed in Table 5. As shown in Table 5, the two-body decomposition energies and the corresponding energy barriers (ΔE_TS1_) calculated by MP2(full) method are very close to the CCSD(T) results. However, such consistence does not be reproduced for the three-body channels, especially the MP2(full) overestimates the energy barrier (ΔE_TS2_) of the three-body channel over 20 kcal·mol^−1^ with respect with that of CCSD(T), which is due to that MP2(full) method is inadequate to describe the electronic dynamics correlations in HNgCCX system although the true transition state (TS2) for three-body channel obtained by MP2(full) method was confirmed by IRC calculation. Therefore, the discussions below mainly focus on the CCSD(T) energies since CCSD(T) has been accepted as the “golden criterion”.

**Table 5:** The decomposition energies (in kcal·mol-1) of HNgCCX (Ng = Kr and Xe; X = F, Cl, Br and I) and the energy barriers (in kcal·mol^−1^) of the concerning transition states calculated at the MP2(full) and CCSD(T) levels^a^.

	MP2				CCSD(T)			
	ΔE_2B_	ΔE_3B_	ΔE_TS1_^b^	ΔE_TS2_^c^	ΔE_2B_	ΔE_3B_	ΔE_TS1_^b^	ΔE_TS2_^c^
HKrCCF	−124.7	17.2	36.8	25.8	−126.9	13.6	37.2	4.7
HKrCCCl	−124.0	18.9	36.8	24.0	−126.4	13.5	36.8	4.4
HKrCCBr	−123.5	20.0	36.9	23.4	−126.2	13.6	36.8	4.4
HKrCCI	−122.9	21.2	36.9	22.5	−125.8	13.3	36.6	4.2
HXeCCF	−104.7	37.2	44.3	43.6	−105.7	34.7	45.1	21.6
HXeCCCl	−104.1	38.8	44.2	42.1	−105.4	34.6	44.6	22.5
HXeCCBr	−103.6	39.9	44.2	41.6	−105.1	34.6	44.5	22.4
HXeCCI	−103.2	40.9	44.1	40.6	−104.9	34.2	44.2	21.9

^a^ZPE corrections were in the MP2 energies, and no ZPE corrections were considered in the CCSD(T) energies;

^b^ΔE_TS1_ = E_TS1_ − E_HNgCCX_;

^c^ΔE_TS2_ = E_TS2_ − E_HNgCCX_.

As shown in Table 5, the calculated decomposition energies of HNgCCX (Ng = Kr and Xe; X = F and Cl) keep in line with previous works^4, 14^. The two-body channels of HNgCCX are exothermic, and the corresponding decomposition energies of HKrCCX and HXeCCX (X = F, Cl, Br and I) calculated at the CCSD(T) level are about −126 and −106 kcal·mol^−1^, respectively, so these HNgCCX molecules show thermodynamic instability on two-body channels. On the contrary, HNgCCX is thermodynamic stable with respect to the three-body channel because the three-body channel is endothermic, and the CCSD(T) decomposition energies are about 14 and 35 kcal·mol^−1^ for HKrCCX and HXeCCX, respectively. Moreover, the thermodynamic stability of HXeCCX is obviously higher than that of HKrCCX due to the larger ΔE_3B_. In addition, it is clear that the decomposition energies of HNgCCX was almost unaffected by the replacement of halogen atoms.

The kinetic stability of HNgCCX (Ng = Kr and Xe; X = F, Cl, Br and I) was also investigated by calculating the energy along the stationary points on the path of each channel. The models of transition states were shown in Fig. 1, the relevant geometrical parameters of these transition states were listed in Figs S2 and S3, respectively, their harmonic vibrational frequencies were given in Table S1, and the IRC results were provided in Figs S4 and S5, respectively (see Supplementary information). As shown in Figs S4 and S5, the IRC calculations confirm that the transition states connect the appropriate minima. It should be noted that for some HXeCCX systems TS2 seems is higher than TS1 for two reasons, one is that the IRC energies were performed at the MP2(full) level which overestimates the energy barrier (ΔE_TS2_) of the three-body channel with respect with CCSD(T), and the other is that no ZPVE corrections were not considered during calculations. The transition state TS1 for the two-body channel of HNgCCX has a bending H-Ng-C angle of about 100.0°, and the Ng-C bond is elongated and the H-Ng bond is shortened simultaneously with respect to the equilibrium structure of HNgCCX. The imaginary frequency values associated with TS1 involving the bending coordinate are within the ranges of 610~580 and 625~600 cm^−1^ for HKrCCX and HXeCCX, respectively. The corresponding barrier heights calculated at the CCSD(T) level are about 37 and 45 kcal·mol^−1^ for HKrCCX and HXeCCX, respectively, which indicates that HNgCCX is kinetically stable with respect to the bending reaction coordinate, and the kinetically stability of HKrCCX is less than its Xe analogues.

The transition state (TS2) for the three-body channel of HKrCCX has similar structure to the optimized HKrCCX, and the main difference is that the H-Kr and Kr-C bonds are elongated simultaneously. The imaginary frequency values associated with TS2 involving the stretching coordinate is within 719~704 cm^−1^, and the corresponding barrier heights (ΔE_TS2_) calculated at the CCSD(T) level is about 4 kcal·mol^−1^, which indicates that HKrCCX is kinetically stable with respect to the three-body channel as well. Moreover, the three-body channels of HKrCCX is the main decomposition channel because the ΔE_TS2_ is lower than that of TS1 remarkably. Unlike the TS2 of HKrCCX, the TS2 of HXeCCX is one planar structure rather than linear structure, the H-Xe and Xe-C bonds are elongated simultaneously, and the ∠Xe-C-C is within the range of 115~119°. The concerning imaginary frequency of TS2 in Xe systems is within the range of 760~755 cm^−1^, and the corresponding barrier heights (ΔE_TS2_) is about 22 kcal·mol^−1^, which illustrates that HXeCCX has higher kinetically stabilities with respect to the three-body channel than that of HKrCCX. the main decomposition channel of HXeCCX is also the three-body channels is because the ΔE_TS2_ is barely half of the height of TS1. In summary, although HNgCCX is instable thermodynamically with respect to Ng + HCCX, it is kinetically stable with respect to the two-/three-body channels due to the relatively larger energy barriers.

In conclusion, HNgCCX has two decomposition channels (including two- and three-body channels). HNgCCX is kinetically stable with respect to the two-/three-body channels because of the relatively larger energy barriers, and the three-body channels of HNgCCX is the main decomposition channel. HXeCCX is more stable kinetically than its Kr analogues due to the higher energy barriers (ΔE_TS1_ and ΔE_TS2_). The planar TS2 of HXeCCX is different from the TS2 of HKrCCX which is linear. In addition, MP2(full) method is inadequate to describe the three-body channel of HNgCCX although the true TS2 obtained by MP2(full) method was confirmed by IRC calculation, and such reaction mechanism require more accurate multi-configuration interaction methods.

## Conclusions

HNgCCX (Ng = Kr and Xe; X = F, Cl, Br and I) has been investigated by ab initio calculations. Equilibrium geometry, harmonic vibrational frequencies, energies were calculated, and AIM as well as FBO analyses were also carried out to deepen the knowledge of the bonding interactions in the studied molecules.

1. The *v*_H-Ng_ with the highest intensity is the characteristic absorption peak of HNgCCX, and decreasing of *v*_H-Ng_ illustrates that the H-Ng bond is weakened as X varies from F to I. moreover, the H-Xe bond is stronger than the H-Kr bond due to the higher *v*_H-Xe_.
2. Compared with the *v*_C≡C_ in the precursor HCCX, the *v*_C≡C_ in HNgCCX exhibits red-shift character, which indicates that the C≡C bond is activated by insertion of Ng atoms into the H-C bonds of HCCX. The C≡C bond of HNgCCF is expected to be the strongest, and is weakened as X varies from F to I.
3. The *v*_C-X_ in HNgCCX exhibits blue shift of tens wavenumbers with respect to the precursor HCCX, however, the C-X bond is not the general sense of blue-shift halogen bond because it is lengthened during the insertion of Ng atoms into the H-C bonds of HCCX. Moreover, the *v*_C-X_ is mainly affected by the replacement of halogen atoms rather than the replacement of noble-gas atoms.
4. AIM analyses illustrates that the H-Ng bond is essentially covalent bond. However, the covalent character of H-Xe bond is underestimated so that the H-Xe bond behaves like polar ionic bond to a certain extent. There is a gradually transition of C-X bond from covalent bond to ionic bond, and the trend of the covalent character is C-Cl > C-Br > C-F > C-I.
5. FBO calculations shows that both H-Kr and Kr-C bonds in HKrCCX are weaker than the corresponding H-Xe and Xe-C bond in its Xe analogues, but the C≡C bond in HKrCCX is stronger that the corresponding one in HXeCCX, which illustrates that the H-C bond of HCCX is easier to react with Xe atom rather than Kr atom.
6. Although HNgCCX is instable thermodynamically with respect to Ng + HCCX, it is kinetically stable with respect to the two-/three-body channels due to the relatively larger energy barriers. The three-body channels of HNgCCX is the main decomposition channel, and the kinetically stability of HXeCCX is more than its Kr analogues. Of note is that the TS2 of HXeCCX is one planar structure which is different from the linear structure of the TS2 of HKrCCX.

## Computational Methods

All ab initio calculations were carried out using the Gaussian09 program^42^. The augmented Dunning’s correlation consistent valence triple-zeta (aug-cc-pVTZ) basis sets^43, 44^ were used for H, C, F and Cl atoms. To reduce the computation times and consider relativistic effect of heavy elements, the aug-cc-pVTZ-PP small-core relativistic effective core potential (RECP) together with their corresponding basis sets^45, 46^ were used for all other atoms, and the RECP retains 25 and 26 explicit electrons for halogen atoms (Br and I) and noble-gas atoms (Kr and Xe), respectively. HNgCCX (Ng = Kr and Xe; X = F, Cl, Br and I) and the concerning transition states (TS1 and TS2) were firstly optimized by the second-order Møller–Plesset perturbation theory (MP2(full))^47^, then the harmonic vibrational frequency calculation using analytic second derivatives to check that the optimized structure is the minima or true transition states, and the zero-point vibrational energies (ZPVE) were calculated simultaneously, moreover, intrinsic reaction coordinate (IRC) calculations were carried out by MP2 method to verify that the transition states connect the appropriate minima. To obtain more accurate energy of HNgCCX and the concerning transition states, the CCSD(T) single-point energy over the optimized geometry was calculated using the same RECP and basis sets.

To fully understand the nature of the bonding interactions in HNgCCX molecules, Bader’s atoms-in-molecule (AIM)^48, 49^ and fuzzy bond orders (FBO)^35, 36^ calculations were performed by the Multiwfn software^50^. To avoid the negative effects of diffuse functions, the cc-pVTZ/cc-pVTZ-PP basis sets removing diffuse functions were used to FBO calculation. All-electron basis sets rather than ECP basis sets mentioned above were used to generate wave functions for AIM analyses to obtain reasonable results in chemical sense, for example, the WTBS basis set^51, 52^ was used for I and Xe atoms, and the aug-cc-pVTZ basis set for other atoms.

## Electronic supplementary material

Supplementary information
