# Supplementary material for: An ab initio study on noble gas inserted halogenated acetylene: HNgCCX (Ng = Kr and Xe; X = halogen)
Source: Sci Rep. 2017 Aug 31;7:10278. doi: 10.1038/s41598-017-10786-0 (PMC5579194; doi:10.1038/s41598-017-10786-0)
Supplement: Supplementary file 1 — Supplementary information [file 41598_2017_10786_MOESM1_ESM.pdf]

# Supplementary information

## An ab initio study on noble gas inserted halogenated acetylene: HNgCCX (Ng = Kr and Xe; X = halogen)

Zhengguo Huang[*], Yuying Li & Xiaohong Wang

*Tianjin Key Laboratory of Structure and Performance for Functional Molecules; Key Laboratory of Inorganic-Organic Hybrid Functional Materials Chemistry (Tianjin Normal University), Ministry of Education; College of Chemistry, Tianjin Normal University, Tianjin 300387, People's Republic of China*

[*] Correspondence and requests for materials should be addressed to Z. G. Huang (E-mail: hsxyhzg@126.com)

Table S1. The harmonic vibrational frequencies (in cm-1) of transition states (TS1 and TS2) for the two-/three-body channels of HNgCCX (Ng = Kr and Xe; X = F, Cl, Br and I) calculated at the MP2(full)/aug-cc-pVTZ-PP/aug-cc-pVTZ level [a].

| Molecule | $v_1$ | $v_2$ | $v_3$ | $v_4$ | $v_5$ | $v_6$ | $v_7$ | $v_8$ | $v_9$ | $v_{10}$ |
|---|---|---|---|---|---|---|---|---|---|---|
| | | | | | | TS1 (HNgCCX → Ng + HCCX) | | | | |
| HKrCCF | -609.6 (8) | 52.5 (40) | 60.2 (35) | 196.0 (61) | 374.7 (0) | 374.7 (0) | 1008.2 (53) | 2070.0 (171) | 2676.7 (231) | |
| HKrCCCl | -604.0 (6) | 37.3 (37) | 46.3 (46) | 171.7 (50) | 312.7 (0) | 314.5 (0) | 744.2 (1) | 1982.8 (45) | 2674.4 (232) | |
| HKrCCBr | -602.8 (5) | 40.2 (37) | 46.5 (60) | 145.1 (36) | 281.7 (0) | 283.3 (0) | 619.3 (19) | 1963.8 (23) | 2673.8 (231) | |
| HKrCCI | -580.5 (9) | 49.2 (36) | 55.7 (39) | 126.4 (26) | 253.3 (0) | 255.9 (0) | 554.1 (52) | 1944.7 (4) | 2676.2 (242) | |
| HXeCCF | -625.2 (9) | 26.3 (32) | 37.8 (32) | 179.6 (71) | 374.1 (0) | 377.8 (0) | 1010.3 (41) | 2063.2 (243) | 2449.0 (124) | |
| HXeCCCl | -604.4 (12) | 26.8 (29) | 36.1 (18) | 152.7 (55) | 314.4 (0) | 316.5 (0) | 742.1 (4) | 1971.7 (83) | 2447.4 (130) | |
| HXeCCBr | -605.1 (10) | 31.2 (30) | 38.3 (31) | 124.8 (36) | 282.4 (0) | 285.0 (0) | 617.2 (29) | 1955.7 (52) | 2447.9 (129) | |
| HXeCCI | -600.1 (10) | 27.3 (29) | 36.7 (29) | 110.3 (27) | 246.8 (0) | 248.4 (0) | 556.0 (70) | 1943.9 (22) | 2447.2 (130) | |
| | | | | | | TS2 (HNgCCX → H + Ng + CCX) | | | | |
| HKrCCF | -718.7 (45) | 24.4 (2) | 24.4 (2) | 159.2 (3) | 159.2 (3) | 245.4 (158) | 257.1 (4) | 257.1 (4) | 1071.7 (242) | 2268.3 (186) |
| HKrCCCl | -712.4 (54) | 42.6 (1) | 42.6 (1) | 167.5 (3) | 167.5 (3) | 203.6 (1) | 203.6 (1) | 229.0 (163) | 777.5 (275) | 2112.0 (108) |
| HKrCCBr | -708.4 (50) | 38.1 (1) | 38.1 (1) | 167.9 (3) | 167.9 (3) | 171.8 (0) | 171.8 (0) | 194.1 (115) | 648.1 (349) | 2078.9 (95) |
| HKrCCI | -704.2 (51) | 28.8 (1) | 28.8 (1) | 130.4 (0) | 130.4 (0) | 172.4 (3) | 172.4 (3) | 174.5 (97) | 592.8 (408) | 2043.6 (96) |

| | | | | | | | | | |
|---|---|---|---|---|---|---|---|---|---|
| HXeCCF | -760.7 (88) | 60.9 (9) | 109.7 (1) | 112.1 (0) | 179.3 (170) | 350.4 (1) | 429.2 (41) | 1019.0 (167) | 1880.4 (462) |
| HXeCCCl | -764.5 (154) | 41.1 (7) | 112.6 (0) | 117.8 (0) | 171.3 (204) | 283.4 (0) | 376.2 (50) | 760.2 (134) | 1787.2 (1030) |
| HXeCCBr | -759.3 (159) | 33.7 (5) | 113.0 (0) | 118.6 (0) | 157.3 (175) | 250.3 (0) | 348.3 (112) | 624.0 (169) | 1752.5 (1050) |
| HXeCCI | -754.9 (144) | 31.4 (5) | 114.9 (0) | 121.2 (0) | 144.3 (141) | 210.6 (0) | 326.2 (229) | 558.8 (252) | 1727.6 (980) |

[a] Numbers given in parentheses are the respective calculated IR intensities (in km·mol$^{-1}$).

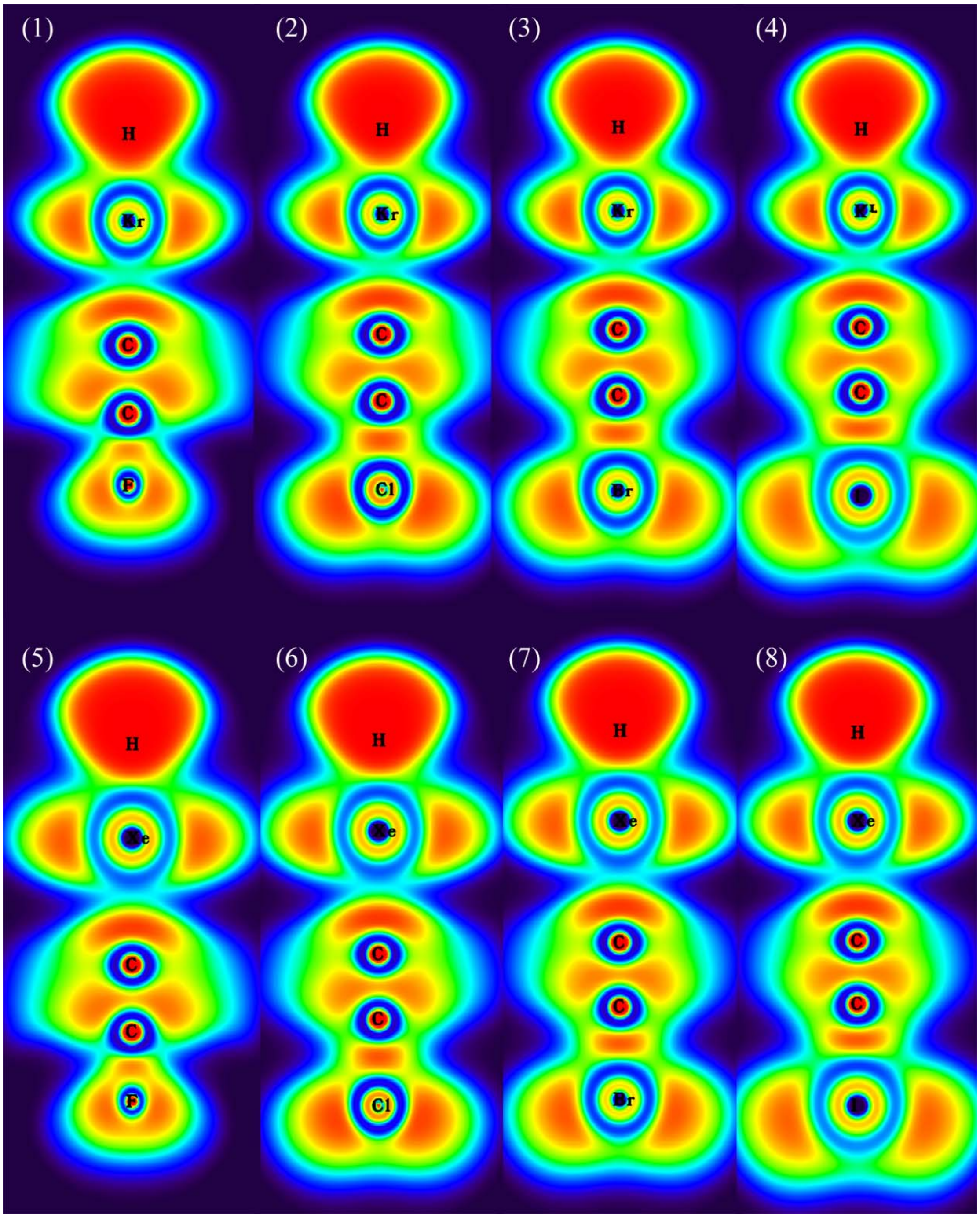


Fig. S1. The ELF color-filled map of HNgCCX (Ng = Kr and Xe; X = F, Cl, Br and I) obtained by MP2(full) method with all-electron basis sets. (1) HKrCCF; (2) HKrCCCl; (3) HKrCCBr; (4) HKrCCI; (5) HXeCCF; (6) HXeCCCl; (7) HXeCCBr; (8) HXeCCI.

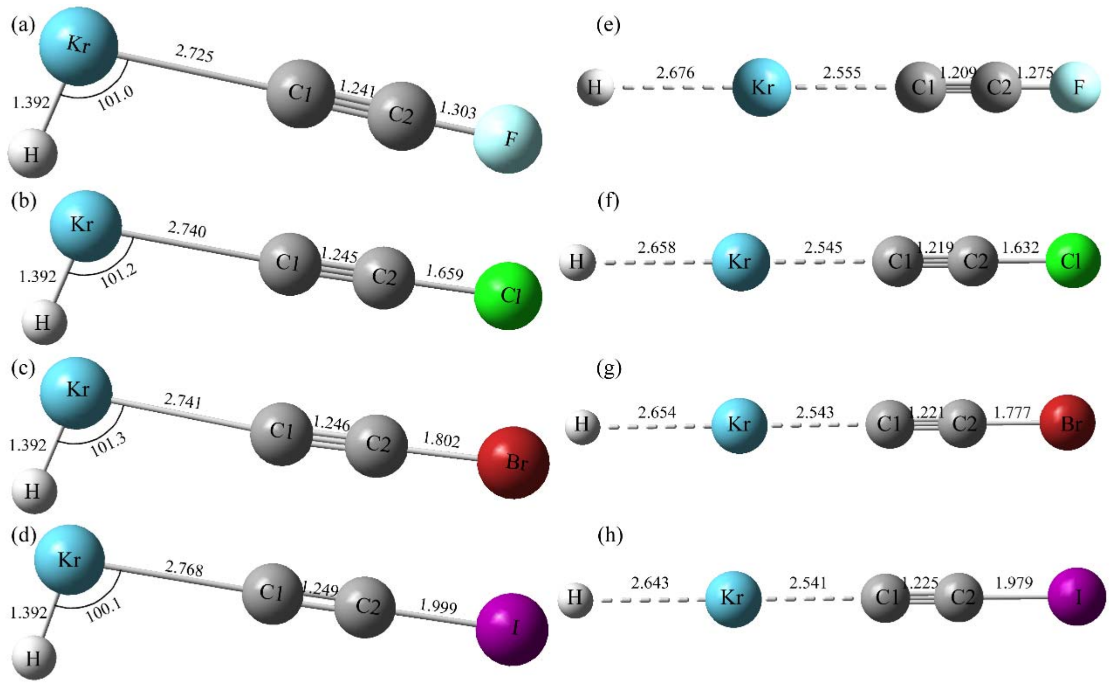


Fig. S2. The structures of the transition states (TS) concerning the two-/three-body channels of HKrCCX (X = F, Cl, Br and I) calculated at the MP2(full) level. (a) ~ (d): TS1 for the two-body channels; (e) ~ (h): TS2 for the three-body channels.

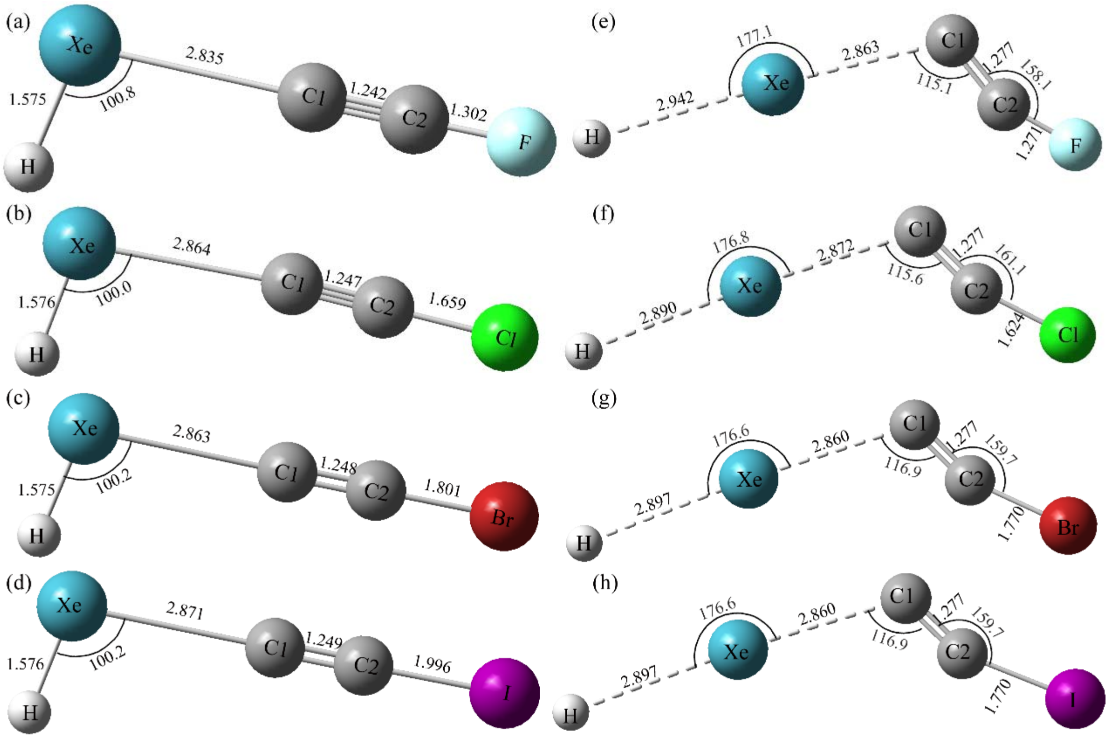


Fig. S3. The structures of the transition states (TS) concerning the two-/three-body channels of HXeCCX (X = F, Cl, Br and I) calculated at the MP2(full) level. (a) ~ (d): TS1 for the two-body channels; (e) ~ (h): TS2 for the three-body channels.

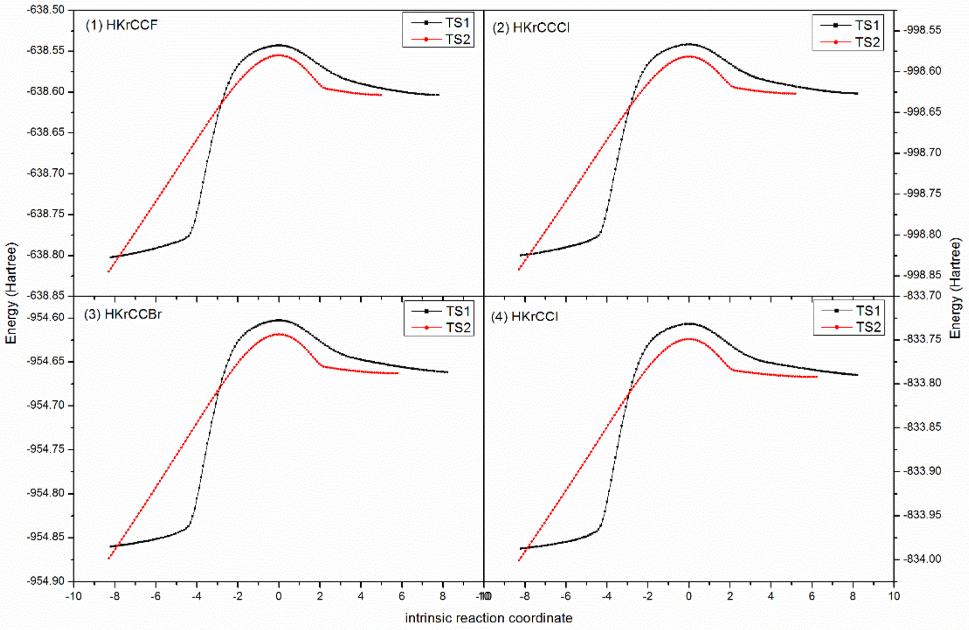


Fig. S4. The IRC results concerning the two-/three-body channels of HKrCCX (X = F, Cl, Br and I) calculated at the MP2(full) level.

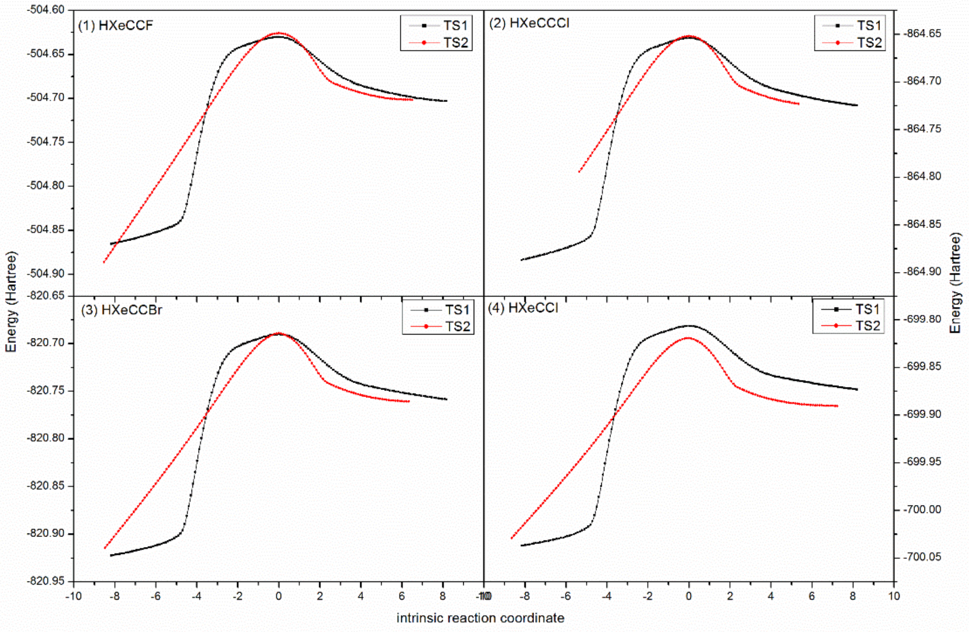


Fig. S5. The IRC results concerning the two-/three-body channels of HXeCCX (X = F, Cl, Br and I) calculated at the MP2(full) level.
